# Treatment Modalities for Acne

**DOI:** 10.3390/molecules21081063

**Published:** 2016-08-13

**Authors:** Lizelle Fox, Candice Csongradi, Marique Aucamp, Jeanetta du Plessis, Minja Gerber

**Affiliations:** Centre of Excellence for Pharmaceutical Sciences, North-West University, Private Bag X6001, Potchefstroom 2520, South Africa; Lizelle.Fox@nwu.ac.za (L.F.); candice.csongradi@gmail.com (C.C.); Marique.Aucamp@nwu.ac.za (M.A.); Jeanetta.DuPlessis@nwu.ac.za (J.P.)

**Keywords:** *Acne vulgaris*, acne treatment, topical, systemic, physical therapies, natural

## Abstract

Acne is a common inflammatory skin disease which affects the pilosebaceous units of the skin. It can have severe psychological effects and can leave the patient with severe skin scarring. There are four well-recognized pathological factors responsible for acne which is also the target for acne therapy. In this review, different treatment options are discussed, including topical (i.e., retinoids, and antibiotics) and systemic (i.e., retinoids, antibiotics, and hormonal) treatments. Since the general public has been showing an increasing interest in more natural and generally safer treatment options, the use of complementary and alternative medicines (CAM) for treating acne was also discussed. The use of physical therapies such as comedone extraction, cryoslush therapy, cryotherapy, electrocauterization, intralesional corticosteroids and optical treatments are also mentioned. Acne has been extensively researched with regards to the disease mechanism as well as treatment options. However, due to the increasing resistance of *Propionibacterium acnes* towards the available antibiotics, there is a need for new treatment methods. Additionally, the lack of necessary evidence on the efficacy of CAM therapies makes it necessary for researchers to investigate these treatment options further.

## 1. Introduction

*Acne vulgaris* is a common chronic inflammatory disease of the skin. It is found in about 80% of young adults and adolescents. It is a disease that affects the pilosebaceous units of the skin and may result in inflammatory or non-inflammatory lesions [1,2,3]. Strauss et al. [4] defined acne as a chronic inflammatory dermatosis which consists of open comedones (blackheads), closed comedones (whiteheads) and inflammatory lesions such as nodules, pustules and papules. Thiboutot et al. [5] suggested that acne should be recognized as a chronic disease which may also affect the patient psychologically.

In recent years, acne has been observed in younger patients due to the earlier onset of puberty [6]. Adebamowo et al. [7] stated that acne is more common in girls in the age range of 12 years and younger, but it presents more in boys in the age range of 15 years or older. In most cases, acne disappears within the patient’s early twenties; however, acne may persist into adulthood which usually occurs more often in females [1].

Acne has many negative effects on young adolescents. It causes discomfort, emotional stress, disfigurement and even permanent scarring to the skin. It may also cause anxiety and embarrassment in patients and may diminish the patient’s physiological and social wellbeing [8,9]. Several factors may induce acne production or increase its severity. Some of these factors include genetics, the male sex, youth, stress and smoking as well as comedogenic medications such as androgens, halogens, corticosteroids and pore clogging cosmetics. Past research suggests that genetic influence combined with comedogenic hormones (especially androgens) produce abnormal volumes of sebum which contribute to acne lesions [1,3,10].

At present, there is a widespread interest in the relationship between diet and *Acne vulgaris* [11]. This relationship will, however, not be discussed in the current article as there is a great deal of information available and forms a subject on its own. Diagnosing acne is simple and straightforward. Differential diagnosis that exists is rosacea (lacks comedones), folliculitis, dermatitis and drug-induced eruptions [10].

## 2. Pathogenesis of Acne

Acne affects the pilosebaceous units of the skin which presents with a variety of lesions at various inflammatory stages, including acne scars and hyperpigmentation [10,12]. According to Olutunmbi et al. [10], acne lesions are most commonly present on the face, chest, upper back and upper arms which are known to have a high density of sebaceous glands [6].

**Figure 1:**
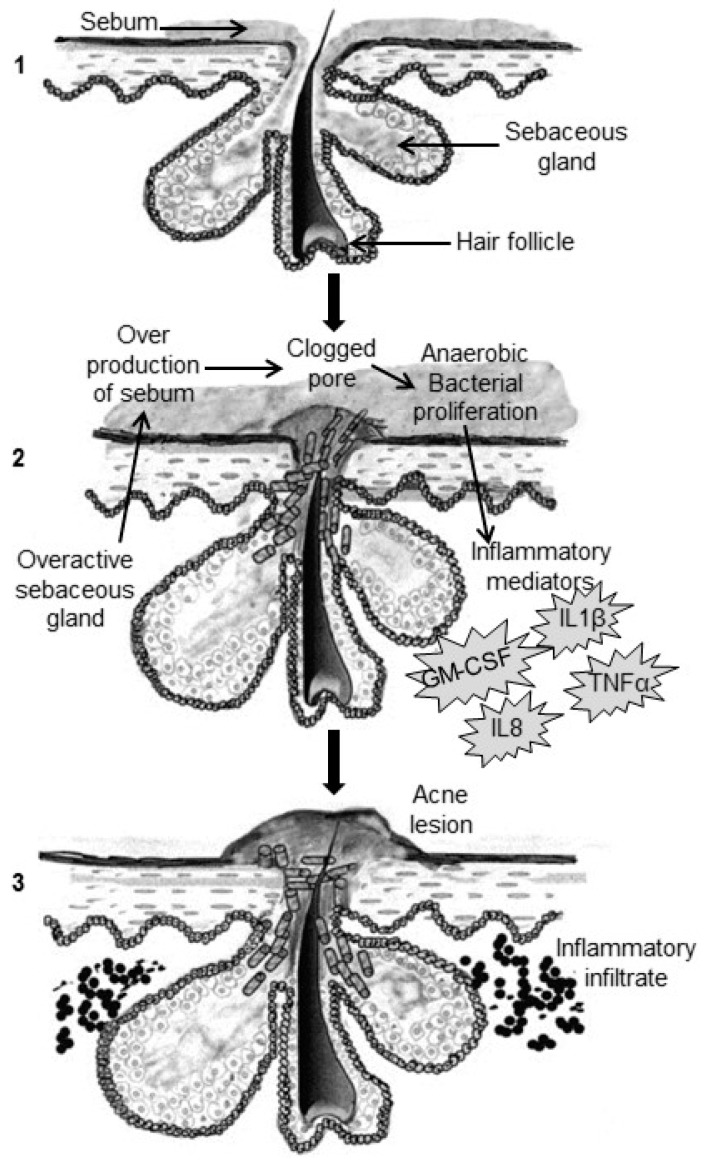
Pathogenic factors contributing to the development of acne: (1) The normal pilosebaceous unit. (2) The clogging of the pore is aggravated by hyperkeratinization and excess sebum production whilst anaerobic bacteria (mainly *P. acnes*) proliferate and inflammatory mediators are released. (3) Inflammatory infiltrates cause the development of increasing degrees of severity in inflammatory acne forms (Reprinted from Drug Discovery Today: Disease Mechanisms, 5, Muizzuddin et al. [14], Acne–a multifaceted problem, e184–e188, Copyright (2008), with permission from Elsevier).

The four main pathological factors involved in the development of acne are the increased sebum production, irregular follicular desquamation, *Propionibacterium acnes* proliferation and inflammation of area [13]. These four factors are illustrated in Figure 1.

### 2.1. Excess Sebum Production

Gollnick [15] stated that androgen hormones (especially testosterone) stimulate increased production and secretion of sebum. Increased sebum production directly correlates with the severity and occurrence of acne lesions and for this reason it is an important factor that should be taken into consideration when dealing with patients suffering from *Acne vulgaris* [15,16].

### 2.2. Epidermal Hyper-Proliferation and Formation of Comedones

The keratinocytes in normal follicles are usually shed to the lumen as single cells which are then excreted. In patients with acne, hyper-proliferation of the keratinocytes occur and they are not shed as they should be, which results in the gathering of the abnormal desquamated corneocytes in the sebaceous follicle along with other lipids and monofilaments. This phenomenon results in comedogenesis [13].

Webster [17] refers to a microcomedone as the first microscopic lesion that forms from occlusion of the follicle, and it is the precursor of the other acne lesions. The microcomedone gradually fills up with more lipids and monofilaments and develops into visible non-inflammatory comedones and inflammatory acne lesions [13,15,18]. Comedones are referred to as blackheads (open comedones) when they are dilated at the skin surface. They appear blackish on the skin and are filled with sebum and desquamated keratinocytes. They can also be termed as whiteheads (closed comedones) which appear as a white bump underneath the skins surface with no open pores. If sebum continues to accumulate, the closed comedone will continue to expand and may rupture into the surrounding tissue [15].

### 2.3. Propionibacterium Acnes Infiltration

The microflora present in a normal sebaceous follicle is qualitatively similar to that found in comedones. This includes three coexisting groups of bacteria, namely (1) coagulase-negative staphylococci (*Staphylococcus epidermidis*); (2) anaerobic diphtheroids (*P. acnes* and *Propionibacterium granulosum*) and (3) lipophilic yeasts (*Pityrosporum* species) [19].

*P. acnes* and *S. epidermidis* differ in their potential to provoke local skin inflammation and to generate pro-inflammatory mediators. It was however determined that *S. epidermidis* is not likely to partake in the pathogenesis of inflammatory *Acne vulgaris* skin lesions as the antibody response to *S. epidermidis* was somewhat harmless compared to the antibodies generated by *P. acnes* [20]. As *S. epidermidis* is an aerobe organism and their growth site is superficial, it is incapable of residing in the anaerobe environment of the infra-infundibulum where the inflammatory process occurs [19]. The lipophilic yeasts present in the pilosebaceous unit do not seem to play a noteworthy etiologic part in any disease conditions [19].

*P. acnes* is an anaerobic, gram positive pathogen that colonizes in sebaceous follicles. It is generally more prevalent in areas of the skin that are densely packed with sebaceous follicles because these follicles produce large volumes of sebum that provide a lipid-rich, anaerobic environment that is optimal for *P. acnes* [15]. It is evident that all individuals have *P. acnes* present on the surface of the skin which can contribute to follicular clogging, but not all individuals present with acne due to the differences in individual immune response to the pathogen [21]. According to McInturff and Kim [22], *P. acnes* produces a lipase enzyme that metabolizes the triglycerides of sebum into glycerol and fatty acids, which may in turn assist in the formation of comedones and the inflammation that follows. *P. acnes* appears to be the most probable organism to cause *Acne vulgaris* and is therefore the target of oral and topical antibiotic treatments [19].

### 2.4. Inflammation Process

The inflammatory process begins when *P. acnes* is detected by the immune system. *P. acnes* has a highly inflammatory effect which may trigger the release of chemostatic factors such as lymphocytes, neutrophils and macrophages. These factors may cause follicular damage, rupture and leakage of bacteria, fatty acids and lipids into the surrounding dermis. This process will give rise to inflammatory lesions (pustules, nodules, cysts and papules). Inflammatory lesions are filled with pus and are larger than non-inflammatory lesions [9,12,13,15]. Additionally it was found that neutrophils generate reactive oxygen species (ROS) which partially contributes to acne inflammation by damaging the follicular epithelium. This leads to the expulsion of the follicular content into the dermis which consequently causes various inflammatory processes [23].

## 3. Current Treatment of Acne

The main goal of acne treatment is to control and treat existing acne lesions, prevent permanent scarring as far as possible, limit the duration of the disorder and to minimize morbidity. The patient should be informed on the aims involved in preventing new acne lesions while allowing the existing ones to heal. Patients should also be made aware that it may take 3–6 weeks until an improvement can be observed [6,9,21].

Individual patient factors must be taken into account when determining a regimen for the treatment of acne. Some of these factors are the current medical condition, disease state, severity of the lesions, endocrine history and the preferred treatment of the patient (oral or topical). Acne may be treated topically or systemically (with oral drugs) as seen in Table 1. Other treatment options include the use of natural products or the use of non-drug treatments, such as for example optical therapy. However, a combination treatment that targets more than one of the mechanisms of acne pathogenesis is often successful. The response of the patient is recorded and the regimen can be adjusted as the clinical condition improves [10].

### 3.1. Topical Treatment

Topical products have the advantage of being applied to the affected area directly; thus decreasing systemic absorption and increasing the exposure of the pilosebaceous units to the treatment. However, a major side effect of topically applied anti-acne products is skin irritation. Preparations for topical application are available as various formulations, including creams, gels, lotions, solutions and washes [24].

Topical therapy is based on the type and severity of acne. Mild acne is often treated with topical retinoids, or a variety of diverse treatments such as azelaic acid, salicylic acid and benzoyl peroxide. Mild to moderate inflammatory acne can be treated with topical anti-inflammatory agents as well as topical antibiotics [9]. The different topical anti-acne drugs target different pathophysiological factors [25] and a few of the common topical treatments will be discussed below.

#### 3.1.1. Retinoids

Topical retinoids can be used as monotherapy for inflammatory acne, in combination with more severe forms of acne or as a maintenance treatment. They generally control the formation of microcomedones, reduce the formation of lesions and existing comedones, decrease sebum production and normalize desquamation of the epithelium. They target the microcomedones and suppress comedone formation. They may also show anti-inflammatory properties [13].

Gollnick and Krautheim [25] gave the following suggestions on the use of topical retinoids: (1) the use of topical retinoids is vital for maintenance treatment; (2) retinoids can repair the scarring and hyperpigmentation of the skin; (3) this class of drugs should be first choice of treatment for most of the acne types; and (4) when combined with topical antimicrobials it is more effective in inflammatory acne. A common side effect during the first few weeks of topical retinoid treatment is a flare up of acne. This should, however, clear as the patient continues with the treatment [26]. Only some of the most common topical retinoids (i.e., tretinoin, adapalene and tazarotene) used in acne treatment will be discussed.

##### Tretinoin

Tretinoin is a form of vitamin A [8]. It is a standard comedolytic agent used in acne treatment to regularize desquamation of the epithelium, which prevents blockage of pilosebaceous units. It also seems to have anti-inflammatory properties. It has been a topical treatment for acne for over three decades [9,27].

##### Adapalene

Adapalene is a synthetic retinoid analogue which is most commonly used as a first line topical retinoid treatment for *Acne vulgaris*. It normalizes the cell differentiation of the follicular epithelium and prevents comedone formation. It also shows anti-inflammatory action on the acne lesions [1,8,9].

##### Tazarotene

Tazarotene is a synthetic acetylenic pro-drug which is converted to tazarotenic acid in keratinocytes [8]. It is one of the newer retinoids used for acne treatment. It affects the keratinocyte differentiation and proliferation in the epithelial tissue and may also show anti-inflammatory properties [15]. It is regarded as a second line treatment after no response was observed after previous tretinoin or adapalene treatment, as it may cause skin irritation in acne patients [9].

##### Other Retinoids

Other retinoids used for topical treatment of acne include isotretinoin, retinoyl β-glucuronide and motretinide [25,26]. However, according to Zaenglein [26], these topical retinoid formulations are unavailable in the USA, although they are commonly used in the European Union. Of these three retinoids, only isotretinoin is available as a topical formulation in South Africa.

#### 3.1.2. Antibiotics

Topical antibiotics are generally used for mild to moderate inflammatory acne. They have activity against *P. acnes*, and therefore act on the surface of the skin to reduce the stimulus for inflammation of the lesions [17]. Due to certain side effects and lesser effectiveness of topical chloramphenicol and tetracyclines, these drugs are less frequently used [25]. The most popular topical antibiotics used in acne treatment are erythromycin and clindamycin, but, in recent years, the continuous use of these antibiotics has led to the increasing development of resistance against *P. acnes* strains [28].

Therefore, it is recommended that monotherapy with topical antibiotics are used for only a short time period (12 weeks) and that the antibiotics should be combined with benzoyl peroxide, zinc or retinoids to prevent bacterial resistance [6,25]. The use of oral and topical antibiotics in combination to treat acne should be avoided [6].

##### Erythromycin

Erythromycin is a macrolide antibiotic that attaches to the 50S ribosomal unit of bacterium and prevents translocation, which is necessary for protein synthesis of the bacteria [8]. It is active against *P. acnes* and reduces the colony on the surface of the skin and within follicles. It has been regarded as a very effective topical antibiotic in acne therapy, but recently it was discovered that erythromycin is up to 60% resistant to *P. acnes* which makes it less desirable. This has led to interest in the future development of other topical antibiotics [27,29].

##### Clindamycin

Clindamycin is classified as a lincosamide antibiotic. It is a semi-synthetic derivative of the antimicrobial agent, lincomycin. Clindamycin attaches to the 50S ribosomal subunit and inhibits protein synthesis of the bacteria and as with erythromycin; it also inhibits *P. acnes* on the surface of the skin [8,27].

#### 3.1.3. Diverse Treatments

Other topical treatments used for acne, such as for example chemical peels, benzoyl peroxide, dapsone, etc. will be discussed in the following section.

##### Salicylic Acid

Salicylic acid is known as a keratolytic agent whose mechanism of action is to dissolve the intercellular cement which holds the cells of the epithelium together [8]. It has a minor anti-inflammatory effect, enhances penetration of certain substances and at low concentrations it is fungistatic and bacteriostatic. Salicylic acid is found in a number of over-the-counter products for acne treatment [8,27].

##### Chemical Peeling with Hydroxy Acids

Chemical peels involve facial resurfacing whereby removal of the epidermis stimulates re-epithelization and skin rejuvenation [30]. Chemical peeling also appears to reduce hyperpigmentation and superficial scarring of the skin [25]. This therapy can be divided into different groups according to its penetration depth and destruction. Alpha-hydroxy acids (i.e., glycolic acid and lactic acid) and beta-hydroxy acids (i.e., salicylic acid) are the most common chemicals used in chemical peels [24,30,31]. A much higher concentration of salicylic acid (20%–30%) is present in chemical peels than found in daily acne cleansers [30]. There exist little evidence/data that peels are relatively safe to use [32]. Therefore, it should be regarded as a complementary treatment rather than a first-line treatment [31].

##### Benzoyl Peroxide

Benzoyl peroxide is a topical disinfectant, originally employed as a peeling agent for treating acne [33]. It possesses diverse properties, making it both a comedolytic and an antibacterial agent, with no effect on sebum production. Benzoyl peroxide has proven bactericidal activity against *P. acnes* by releasing free radical oxygen, which degrades the bacterial proteins [8,27]. Bershad [1] stated that in addition to its successful treatment of inflammatory acne, benzoyl peroxide also decreases the number of comedones on the skin.

Benzoyl peroxide is an important treatment for mild to moderate acne and, although it can be used as monotherapy for a period of 6–8 weeks, is often combined with topical antibiotics in order to reduce the resistance of the *P. acnes* species and to increase the efficacy of treatment [9,15,25]. Gollnick and Krautheim [25] suggested that benzoyl peroxide is best combined with topical retinoids. However, it has been found that all retinoids (except for adapalene) are unstable when combined with benzoyl peroxide and should therefore be applied separately [6]. The main side effects of benzoyl peroxide include burning, dryness, erythema, peeling or stinging [24].

##### Azelaic Acid

Azelaic acid is a natural dicarboxylic acid that inhibits protein synthesis of the *P. acnes* species [34,35]. It is an effective agent because it has bacteriostatic, anti-inflammatory, antioxidant and anti-keratinizing properties [36]. Thusfar, no bacterial resistance of *P. acnes* exists with azelaic acid [37]. It has also been suggested that when azelaic acid is used in conjunction with clindamycin, benzoyl peroxide or α-hydroxy acids it will be more effective [25].

##### Sulfur

In the past, sulfur was frequently used in preparations for acne. This active has, however, become unpopular due to its bad odor [31]. Sulfur is a chemical that has demonstrated to have mild keratolytic and bacteriostatic properties. Sulfur is reduced to hydrogen sulfide inside the keratinocytes which is said to break down keratin in the skin [8]. According to Akhavan and Bershad [8], sulfur also has activity against *P. acnes*.

##### Hydrogen Peroxide

A study by Tung et al. [38] has shown that a regimen based on hydrogen peroxide for treating mild-to-moderate acne compared well with a regimen based on benzoyl peroxide in terms of cosmetic acceptability, efficacy and safety.

##### Niacinamide

Niacinamide is an active amide of vitamin B3 and is composed of niacin (also known as nicotinic acid) and its amide. It may also be referred to as nicotinamide [39,40].Its mechanism of action can be explained as the inhibition of sebocyte secretions, resulting in less sebum production which reduces the oiliness of the skin [40,41]. It also has anti-inflammatory properties which have proved to be beneficial in pustular as well as papular acne [42,43]. Topical application of a 4% niacinamide has led to significant improvements to acne all over the world [40].

##### Topical Corticosteroids

Topical corticosteroids can be used in certain conditions, as for example to treat very inflammatory acne. The treatment period should, however, be short [25] and proof of their efficiency should still be determined [31].

##### Triclosan

Triclosan is an antibacterial agent (antiseptic) which can be used to treat acne [44]. It was determined that bacterial populations did not develop resistance to triclosan under clinical conditions [45]. No adverse effects are anticipated when triclosan containing products are used as recommended [46].

##### Sodium Sulfacetamide

This agent belongs to the sulfonamide antibacterial group. It is bacteriostatic by inhibiting deoxyribonucleic acid (DNA)-synthesis through competitive antagonism of para-aminobenzoic acid (PABA). Sodium sulfacetamide has activity against a number of gram-positive and gram-negative agents, but is generally only used when other topical agents cannot be tolerated by patients [8,9].

##### Dapsone

Dapsone possesses antibacterial and anti-inflammatory activity, although its precise mechanism of action against acne is still unknown [31,47]. However, it has recently been suggested that dapsone’s mechanism of action in the treatment of acne may be due to antimicrobial and anti-inflammatory effects [48]. Dapsone gel (5%) can be used to reduce inflammatory as well as non-inflammatory acne lesions [49]. This agent’s lower cost makes it more favorable for use in developing countries [31]; however, it is not recommended as first-line therapy [47].

### 3.2. Systemic Treatment

Oral systemic treatment is required when acne is resistant to topical treatment or if it manifests as nodular lesions or leaves scarring. It is the preferred choice in the treatment of inflammatory lesions. Systemic treatment may also be required to prevent social embarrassment and psychological impairment in people suffering from acne. The most common systemic treatment includes isotretinoin, oral antibiotics and hormonal agents [12,50].

#### 3.2.1. Retinoids

Isotretinoin is a systemic retinoid and derivative of vitamin A. It is currently being used as a first line treatment for severe nodular or inflammatory acne and is the only known medication which has the potential for the suppression of acne in the long term [9,50]. It can also benefit patients with mild to moderate cases of acne that have proved resistant to topical or other oral agents in the past. It is also considered as a first line treatment in severe acne of the face and trunk, acne that causes scarring and acne that causes psychological complications [50].

Currently, isotretinoin is the only drug available which has an effect on all four pathogenic factors of acne [6,51]. Isotretinoin causes de-differentiation of the sebaceous gland, decreasing the sebum production which will lead to a change in the ecosystem of the cutaneous bacterial flora, ultimately reducing *P. acnes* colonization in the follicles. It also causes the shedding of the keratinocytes [1,9,51,52]. Isotretinoin treatment is normally over a course of 16–24 weeks [6]. It is necessary to closely monitor patients who use isotretinoin because of its harmful side-effects [12].

#### 3.2.2. Antibiotics

Oral antibiotics are generally indicated for moderate to severe inflammatory acne, acne that shows resistance to previous topical treatment or for acne that covers a large surface area of the body [33,50]. Acne is often treated with oral antibiotics such as macrolides (erythromycin, clindamycin, azithromycin and roxithromycin), fluoroquinolones (levofloxacin), tetracyclines (doxycycline, minocycline and lymecycline) and co-trimoxazole [13,51,53].

These antimicrobial agents inhibit the growth of *P. acnes* and the synthesis of inflammatory mediators released from *P. acnes*. The success of the antibiotic treatment is based on the ability of the agent to reach the lipid environment of the pilosebaceous follicles in the dermis, which is the area where *P. acnes* colonize. Tetracyclines are very popular because they are effective and inexpensive. Doxycycline and minocycline are preferred because they cause less gastrointestinal irritation, and they are more lipid soluble, penetrating the pilosebaceous follicle more efficiently [13,50]. The tetracycline family exhibits both anti-inflammatory as well as antibacterial properties. Additionally, less resistance in *P. acnes* have been reported with the tetracyclines than the macrolides [33].

Not many studies have been performed to determine the efficacy of azithromycin in the treatment of acne, whereas clindamycin (topical) and erythromycin (topical and oral) have been well recognized as acne treatments [33]. Erythromycin and clindamycin have little anti-inflammatory activity and mainly work by reducing the levels of *P. acnes* [33].

Since these antibiotics are used repetitively at low doses for extended periods of time [13] during acne treatment, increasing resistance has developed overtime which has resulted in limited use of these agents [13,50]. To reduce resistance and improve the efficacy, oral antibiotics should be combined with topical benzoyl peroxide or retinoids. Additionally, the duration of treatment should not exceed 12 weeks when feasible [6]. It has also been suggested that if a patient is a good candidate for treatment with isotretinoin, long-term antibiotic treatment is unfeasible [33].

#### 3.2.3. Hormonal

Sebaceous glands are androgen dependent and therefore the effect of androgen on sebaceous glands can be treated with hormone therapy [13]. Hormonal treatment can be used as an alternative for adolescent and adult females. These hormones are most commonly given in the form of oral contraceptive pills. The contraceptive hormones reduce the sebum production that is initially induced by androgen. It increases the synthesis of sex hormone-binding globulin which in turn decreases biologically active free testosterone in the female body. Although all contraceptives can be used to treat hormone related acne, progestins are usually preferred because they possess no androgen activity [50].

Oral contraceptives can be used alone or in combination with other therapies to treat acne in women [54]. The treatment period of acne with hormonal anti-androgens must be at least for 12 months and oftentimes even longer [51] as the favorable effect of hormonal agents will only be visible after 3–6 months of treatment [6]. Spironolactone is an alternative drug which can also be combined with oral contraceptives in the treatment of hormone related acne. Its mechanism is based on the fact that it is an androgen receptor blocker. It is especially effective for patients with inflammatory acne [1,50].

#### 3.2.4. Diverse Treatments

Other oral treatments that can possibly be used as adjunctive acne therapy include zinc sulfate, ibuprofen (due to its anti-inflammatory effect) and clofazimine [24,31]. Systemic corticosteroids can be used for initial treatment of inflammatory manifestations (*Acne fulminans*). It can also be used to manage aggravation of acne when treating with systemic isotretinoin [55]. It has been recommended that severe inflammatory *Acne vulgaris*, *Acne fulminans* and *Pyoderma faciale* be treated with oral prednisone (0.5–1.0 mg/kg daily) for a period of 4–6 weeks, after which the dosage can be decreased gradually [31].

### 3.3. Complementary and Alternative Medicines (CAM)

More efficient and safer treatment options are needed for the treatment of acne [56]. Numerous CAM therapies have been noted and/or promoted for use as acne treatment [57] and are generally regarded as safe. Botanical therapies have the added benefit of possessing several modes of action due their composition consisting out of a range of possible active components [58]. It has been proposed that CAM therapies influence the androgenicity, increased sebum activity, infection, inflammation and hyperkeratinization associated with acne [57]. However, in most cases evidence for their use is inadequate [57] and one should still be wary of the possible harm and side effects these plant-derived products can lead to [58]. Some researchers are of the opinion that botanicals may lessen antibiotic resistance when used as alternatives to or in combination with antibiotics. This should, however, still be verified with clinical studies [58].

Various articles [57,58,59] list all the possible plant/herbal remedies for acne. Some of these ingredients do however have some anti-inflammatory, moisturizing and soothing properties. Therefore, theoretically, these ingredients should be able to help relieve some of the drying effects caused by the more vigorous acne therapies and the erythema associated with inflammatory acne [24]. The absence of clinical data on the efficacy of these complimentary remedies is of big concern and needs to be addressed by future research. For the purpose of this article, only a few of the major CAM therapies will be discussed in detail. There is, however, a strong possibility that the range of CAM therapies being used by acne patients is much larger than the series of treatments mentioned in this review [57].

#### 3.3.1. Basil Oil

Advocated topically applied basil essential oils for the treatment of acne include *Ocimum sanctum*, *Ocimum basilicum* and *Ocimum gratissimum* [57]. Since ancient time, Thai basil oils such as *O. basilicum* L. (sweet basil) and *O. sanctum* L. (holy basil) have been used as traditional medicine to treat ringworm and insect bites [60].

Studies showed that topical application of a preparation containing *O. gratissimum* oil in a cetomacrogol blend base were more efficient and reduced lesion counts faster than a 10% benzoyl peroxide lotion [61]. A study on Thai basil oils showed that *O. basilicum* and *O. sanctum* showed promise to be used for acne treatment as they exhibited antimicrobial activity against *P. acnes*. The formulations containing *O. basilicum* showed a higher anti-*P. acnes* activity than the *O. sanctum* containing formulation [60].

#### 3.3.2. Copaiba Oil

Copaiba oil-resin has traditionally been used as an antiseptic, anti-inflammatory and healing agent. Da Silva et al. [62] conducted a double-blind placebo controlled clinical trial in which the copaiba oil was prepared into a topical gel to determine its activity against *Acne vulgaris*. After 21 days of treatment, the copaiba oil gel stopped the outbreak of new pustules, healed pre-existent pustules and reduced the area of erythema. The authors [62] concluded that copaiba oil may be used in the treatment of mild acne, although larger studies are necessary to confirm.

#### 3.3.3. Green Tea

Green tea possesses anti-inflammatory, anti-oxidant, antimicrobial and antimutagenic properties which can be ascribed to its high content of polyphenols, including catechins (flavan-3-ols). The main catechins found in green tea include epigallocatechin-3-gallate (EGCG), epigallocatechin (EGC), epicatechin-3-gallate (ECG) and epicatechin (EC) [63], of which EGCG is the most abundant polyphenol found in green tea [64].

Polyphenon-60 from green tea is a mixture of polyphenolic compounds [65]. Topical application of polyphenon-60 in patients with mild-to-moderate acne (in vivo testing) decreased the average amount of open-comedos and pustules. However, polyphenon-60 showed no improvement on closed-comedos. In vitro studies to determine the underlying mechanism by which polyphenon-60 has this therapeutic effect on acne showed that this compound suppresses the inflammation process [66].

Yoon et al. [67] conducted in vitro studies in which it was determined that EGCG directly targets three pathological processes of acne as it has sebo-suppressive effects, it inhibits the growth of *P. acnes* and it has anti-inflammatory effects. They also found that EGCG may reverse the modified keratinization of follicular keratinocytes associated with acne. These results were followed by a double-blinded, split-face clinical trial which showed that the mean inflammatory and non-inflammatory lesion counts significantly decreased after eight weeks of treatment with an EGCG solution when compared to the baseline values.

Results obtained from a double-blind, placebo-controlled, randomized clinical trial showed that when green tea extract was given orally it was found to be effective against acne lesions in mild-to-moderate acne cases. Compared to the control, the green tea extract significantly decreased the inflamed and total lesion counts, although no significant effect was observed on the non-inflamed lesion count [68]. A topical 3% green tea emulsion was found to decrease the sebum production of the cheeks of healthy male volunteers over a 60 day period [69].

#### 3.3.4. Minerals

Minerals have been used for healing purposes since prehistoric times. The minerals being used for therapeutic intents are mostly clay minerals such as kaolinite, palygorskite, smectites and talc. Clay minerals can be used to treat acne, blackheads and spots. Generally it is applied as a face mask consisting of a warm mixture of water and clay which will open pilosebaceous orifices, stimulate perspiration and sebaceous secretions [70]. A mix of minerals (consisting primarily of halloysite, sericite and talc) obtained from ores indigenous to Korea showed to inhibit the growth of *S. epidermidis* and *P. acnes* [71].

Dead Sea black mud showed marked antimicrobial action when test microorganisms (*P. acnes*) were added to the mud where after they lost their viability. Additionally, when Dead Sea mud was placed on *P. acnes* inoculated agar plates, a growth inhibition zone was observed [72]. Another mineral commonly used both systemically as well as topically for the treatment of *Acne vulgaris* includes zinc [73] which was mentioned earlier.

#### 3.3.5. Antimicrobial Peptides

Natural antimicrobial peptides represent promising therapies for treating acne [49] as they are unlikely to provoke drug resistance in microorganisms [74]. However, some authors have stated that development of peptide-based drug resistance has been proven experimentally, although, when compared to conventional antibiotics it is considered to arise at a much slower pace [75].

A synthetic peptide, derived from epinecidin-1 (from the marine organism *Epinephelus coioides*), have shown bactericidal properties against *P. acnes* by means of destroying its membrane [76]. In another study, antimicrobial peptides derived from ranid frog skins showed a high potency against *P. acnes* [75].

Wang and co-workers [74] purified a snake cathelicidin-derived antimicrobial peptide, cathelicidin-BF, from the venoms of *Bungarus fasciatus*. When tested in vitro it was observed that cathelicidin-BF possessed potential antimicrobial activity against *P. acnes*, comparable to that of the antibiotic, clindamycin. Additionally, this antimicrobial peptide showed some anti-inflammatory effects and inhibited *P. acnes*-induced O_2_^−^ production. All these properties suggested the potential use of cathelicidin-BF for treating *Acne vulgaris*.

#### 3.3.6. Resveratrol

Considering the pathophysiology of acne, the ideal drug should be capable of reducing the inflammatory response as well as inhibiting *P. acnes* [76]. As a result resveratrol is emerging as a new approach in treating acne [49] as it possesses anti-proliferative, anti-inflammatory and *P. acnes* inhibiting properties [77,78,79,80].

Resveratrol is a natural phytoalexin which is produced by certain spermatophytes, such as for example grapes [79]. A single-blind, vehicle-controlled pilot study was performed in which resveratrol (*trans*-isomer) was formulated into a gel with a carboxymethylcellulose base. This formulation was applied on the right side of the face of volunteers with inflammatory *Acne vulgaris* in the facial area for 60 days and compared to the left side of the face on which the control (hydrogel vehicle) was applied. All the volunteers had a noteworthy reduction in pustular lesions and inflammation with an overall noticeable clinical improvement on the side of the face treated with resveratrol. The resveratrol-treated side of the face also showed a significant decrease of macrocomedones and microcomedones when compared to the vehicle-treated side of the face. It seemed as though resveratrol inhibited the keratinocyte hyperproliferation process [79].

Resveratrol was found to inhibit *P. acnes* growth when tested in vitro. It was bacteriostatic at lower concentrations (50 mg/L and 100 mg/L) and bactericidal at the highest concentration tested (200 mg/L). The inhibiting effect of resveratrol compared well with the activity of frequently used acne treatments benzoyl peroxide and erythromycin [77].

#### 3.3.7. Rosa Damascena

Rose water and essential oils are produced from the damask rose plants (*R. damascena* Mill.) in hydro-distillation industries [81]. Rose water can be used for numerous skin problems and due to its pleasing fragrance and beneficial properties; it is a vital ingredient in several cosmetics and body creams [82]. Some authors have suggested the use of *R. damascena* for the treatment of skin disorders such as acne [82]. Rose oil can be utilized as an astringent to tone and clean the skin [83].

*R. damascena* extract has shown antioxidant activity and inhibits lipid peroxidation, similar to α-tocopherol [84]. The hydroalcoholic *R. damascena* extract showed analgesic and anti-inflammatory activity, although the oil of this plant failed to show any such activities [85].

An extract of *R. damascena* petals showed antibacterial activity against *Pseudomonas aeruginosa*, *S. epidermidis* and *Bacillus cereus* [82]. A noticeable antimicrobial activity was observed against *P. acnes* in a study by Tsai et al. [86]. However, the authors suggested that further studies are required to evaluate the beneficial effect of this extract in *P. acnes* treatment.

#### 3.3.8. Seaweed

A double-blind, vehicle-controlled trial showed that mild acne was significantly improved when treated topically with a complex of seaweed-derived oligosaccharide (*Laminaria digitata* or kelp) and 0.1% zinc pyrrolidone. Even though both the treatments reduced the amount of comedones and papules/pustules on the facial area of the volunteers, the active containing treatment was significantly more effective. The sebum production was also reduced by both treatments, although no significant differences were observed between them. Subsequently, the investigators suggested that the complex works by decreasing comedone formation and inflammation in preference to affecting the sebaceous glands pharmacologically. Some preliminary in vitro data indicated that the active suppresses *P. acnes* growth. This in combination with its anti-inflammatory effects may be the reason for the improvement of comedones, papules and pustules in mild acne [87].

Choi et al. [88] evaluated the potential antimicrobial activity of 57 seaweed species commonly found around the coast of South Korea. The methanol extracts of three species, i.e., *Ecklonia kurome*, *Ecklonia cava* and *Ishige sinicola*, showed to be the most promising possible therapeutic agents for *Acne vulgaris* due to their strong anti-*P. acnes* and anti-inflammatory activity. At the moderate doses these extracts were investigated, none of them showed to have any severe toxic effects.

#### 3.3.9. Taurine Bromamine (TauBr)

Taurine bromamine (TauBr) and taurine chloramine (TauCl) are the main haloamines produced by neutrophils and eosinophils at an inflammation site. Both of these haloamines have shown anti-oxidant and anti-inflammatory properties [89]. A study by Marcinkiewicz et al. [90] concluded that TauBr (synthesized) in particular is a promising candidate for treating *Acne vulgaris* topically. At non-cytotoxic concentrations TauBr showed significantly stronger bactericidal activity in vitro compared to TauCl. Additionally, TauBr exhibited selective topical disinfectant properties as *P. acnes* was more susceptible to TauBr than *S. epidermidis* [90]. A double blind pilot study showed that TauBr cream reduced acne lesions of patients with mild to moderate inflammatory facial *Acne vulgaris* in a similar manner toclindamycin gel [89,91].

##### Tea Tree Oil

Tea tree oil is obtained from the Australian tree *Melaleuca alternifolia* and has been shown to have some antimicrobial activity [24]. Tea tree oil products are commonly used by patients self-treating their acne. It has been suggested that the antibacterial and anti-inflammatory activity of the oil adds to its promising clinical performance. Numerous studies have shown that tree tea oil products decrease lesion numbers in patients with mild-to-moderate acne [92].

A double-blind placebo-controlled study by Enshaieh et al. [93] showed that a topical gel containing 5% tea tree oil was effective in treating mild-to-moderate *Acne vulgaris* when compared to the placebo, i.e., the vehicle gel alone. Both inflammatory as well as non-inflammatory lesions were reduced by this topical gel.

A study by Lee et al. [94] showed that tea tree oil isolated from the leaves of the plant and its components (terpinen-4-ol, α-terpineol, terpinolene and α-terpinene) exhibited anti-*P. acnes* activity. They also determined that the major active component present in tea tree oil, namely terpinen-4-ol, was mostly responsible for this essential oil’s antibacterial activity. However, minor components in tea tree oil also added to its efficiency.

##### Other Complementary and Alternative Medicines

There are numerous other herbal remedies which are commonly used for the treatment of acne, such as amaranth, arnica, asparagus, birch, calendula, celandine, chaste tree, coriander, jojoba oil, labrador tea, neem, orange peel, pine, poplar, rhubarb, soapwort, stinging nettle and turmeric [59].

However, the list of CAM therapies goes on as other authors [57] list even more acne therapies, such as topically applied essential oils of *A. millefolium*, bay, benzoin, black cumin, chamomile, *Eucalyptus dives*, geranium, juniper twig, lemon, lemon grass, orange, patchouli, petitgrain, rosemary, safflower oil, sandalwood, sunflower oil, *T. officinale* and thyme. They also list a range of other topical plants/herbs such as: bittersweet nightshade, black walnut, borage, cucumber, duckweed, English walnut, fresh lemon, garlic, grapefruit seeds, oak bark, onion, Oregon grape root, pea, pumpkin, rue, vinegar, vitex and witch hazel. Other ingestible plants/herbs include Brewer’s yeast, burdock root, *C. mukul*, *S. flavescens* and *W. somnifera*. Numerous homeopathic, Indian Ayurvedic therapies and Asian topical therapies were also noted to be used in acne treatment [57].

A lotion containing 2% tea effectively cleared papules and pustules in mild to moderate *Acne vulgaris* [95]. A study on Taiwanese herbal extracts revealed that Du Zhong and yerba mate extracts may possibly be used to treat acne due to their anti-inflammatory and antimicrobial activity against *P. acnes* [86]. Standardized pomegranate rind extract has shown bacteriostatic activity against *P. acnes*, *Staphylococcus aureus* and *S. epidermidis*. This extract also showed anti-inflammatory and anti-allergic properties [96].

Another potential anti-acne treatment is the ethanol extract of *Rhodomyrtus tomentosa* (Aiton) Hassk. leaves (also known as rose myrtle) and its principle compound, rhodomyrtone. Both substances showed antibacterial activity against *P. acnes*. However, the rhodomyrtone showed more noteworthy activity closer to that of erythromycin than the extract [97].

Selvan et al. [98] stated that due to restricted antibacterial drug options and a shortage of vaccines against *Acne vulgaris*, concomitant therapeutic strategies, such as the use of specific antibodies, are needed. Specific polyclonal chicken egg yolk antibodies against *P. acnes* were developed by Revathy et al. [99]. Due to their effectiveness, it was suggested that they can be used in the treatment of acne, although their efficiency should still be confirmed in vivo.

A new concept in the treatment of *Acne vulgaris* and other skin diseases were discussed by Wang et al. [100]. This involved the development of probiotics against *Acne vulgaris* by harnessing the bacterial interference (through fermentation) between *P. acnes* and *S. epidermidis*. Their results indicated that the fermentation of naturally occurring glycerol in the skin, as mediated by skin microorganisms (mostly *S. epidermis*), improved their *P. acnes* inhibitory properties [100].

### 3.4. Physical Treatment

There are several physical treatments available which can be used as adjunctive acne treatment. Henceforth, these therapies can play a major role in the treatment of acne as the pathogenesis of acne becomes more understood and technology improves [30].

#### 3.4.1. Comedone Extraction

Some authors have suggested that this technique can be used concurrently with isotretinoin treatment to treat macrocomedones (comedones larger than 1 mm). No residual scarring should be left if this technique is performed correctly [101]. This mechanical method of extraction involves the following: the lesion should be prepped with alcohol and the epidermis lightly pierced with a large-bore needle or surgical blade. Thereafter, a comedone extractor is used to apply light to medium pressure on top of the lesion until all the contents are forced out [31,101]. Prior to the manual removal of the comedone, enzymatic or mechanical exfoliation can be used to decrease hyperkeratosis. After treatment, the skin should be treated with an anti-inflammatory or antimicrobial agent [102].

#### 3.4.2. Cryoslush Therapy

A slush-like mixture consisting of solid carbon dioxide and acetone can be brushed lightly over the infected skin. This will produce desquamation and erythema [31].

#### 3.4.3. Cryotherapy

Cryotherapy involves the regulated and targeted destruction of diseased skin tissue by applying a substance with a very low temperature. Although liquid nitrogen is the most common cryogen used, there are several other cryogens also available, such as carbon dioxide and nitrous oxide. Different techniques can be used to apply the cryogen, including the cryoprobe, dipstick method or the spot freeze technique. Cryotherapy is generally performed without local anesthesia under aseptic conditions and if performed correctly it should result in extremely good cosmetic results [103].

#### 3.4.4. Electrocauterization

Electrocauterization eradicates comedones by means of generating very low-grade thermal damage [31]. The exact mechanism by which it helps to resolve comedones is, however, unknown [104]. It is thought that electrocauterization works by stimulating the defense mechanism (inflammatory) of the body or it could be that cautery provides a route for the contents of the macrocomedone to be discharged to the skin surface [31,104].

#### 3.4.5. Intralesional Corticosteroids

Intralesional corticosteroid injections reduce the formation of keloid scars and prevent reappearance after surgical removal [105]. This procedure is especially efficient for the treatment of inflammatory nodules. However, it can be painful and possibly cause cutaneous atrophy [106]. The most frequently used corticosteroid is triamcinolone acetonide [103].

#### 3.4.6. Optical Treatments

Optical treatments for acne include laser therapy, light sources and photodynamic therapy [49]. Lasers and light-based therapy is commonly used for the treatment of mild to moderate inflammatory *Acne vulgaris*. It has been found that this type of therapy resolves acne faster, is effective, and has fewer side effects, thereby enhancing patient satisfaction. Numerous light sources are available to treat/improve acne by targeting *P. acnes*, including fluorescent lamps, full spectrum light, green light, violet light, blue metal halide lamps and xenon flash lamps. Another light source is lasers which can target the sebaceous glands (alters structure of glands thermally) or oxyhemoglobin (to improve erythema) [107].

Photodynamic therapy (PDT) functions similar to laser/light-based therapy in that the light energy kills *P. acnes*. Whereas other devices need costly, high-power equipment to produce these lights, PDT makes use of a lower power light source [107]. PDTs efficacy is, however, enhanced by the use of topical agents such as aminolevulinic acid (ALA), methyl-aminolevulinic acid (MAL) or alternative photosensitizing agents [30].

### 3.5. Combination Therapy

Due to the various pathological factors responsible for acne development, the use of multimodal therapy which targets different processes simultaneously has been receiving considerable attention [30,108]. Combination products have been found to be more effective in treating acne than monotherapy [32]. Additionally, the availability of existing and introduction of new fixed combination treatments can increase patient adherence as the treatment for patients can be more personalized [108].

Fixed topical combinations such as benzoyl peroxide/topical antibiotic or retinoid/topical antibiotic can be used [108]. It has also been recommended to combine oral antibiotics (doxycycline more than minocycline or tetracycline) with topical drugs (benzoyl peroxide, azelaic acid, retinoids) to treat moderate to severe acne and less severe inflammatory acne which did not respond to sole topical therapy [55].

By combining systemic treatments in this way, it can bring on a prompt reduction in dose and a sooner discontinuation of oral antibiotics. These combinations can also improve patient compliance, increase the effectiveness of the treatment and decrease the development of bacterial resistance [24,51]. However, it is important to remember that this last named beneficial outcome is only relevant for the benzoyl peroxide contact areas, and not for the gut and other areas [33].

One study did, however, determine that a combination of clindamycin and benzoyl peroxide performed only slightly better than benzoyl peroxide alone. In this case, it is important for prescribers to take into account availability (obtainable over-the-counter versus prescription required), additional costs and risks (i.e., antibiotic resistance) before prescribing these actives simultaneously [109].

Isotretinoin often causes a flare up of acne (known as “pseudo” acne fulminans) and therefore it can be combined with corticosteroids for the most severe inflammatory acne (i.e., abscesses, cysts and nodules) [31].

Physical removal of microcysts, macrocomedones or closed comedones will enhance the therapeutic efficacy of topically applied comedolytic agents [24]. It has also been suggested that benzoyl peroxide and salicylic acid, which have different mechanisms of action, be combined to treat acne due to their complementary effect when used together [23,107].

## 4. Conclusions

Acne is a common inflammatory skin disease which causes much distress to patients constantly suffering from it. It has been researched extensively with regards to the disease itself as well as available and potential treatment options. The target for acne therapy is the four well-known pathogenic factors responsible for this disease state. This current review discussed the different options for treating acne such as topical therapies, systemic therapies, CAM and physical treatments. However, due to the increasing resistance of *P. acnes* towards the available antibiotics [28] and inter-patient differences, further research in this field will always be required. Furthermore, the general public has been opting for more natural products for treating various skin diseases making it necessary to further investigate CAM as possible adjunctive acne therapy.

## Figures and Tables

**Table 1:** Different treatment options for acne.

Treatment Methods	Examples
Topical	Retinoids: adapalene, isotretinoin, motretinide, retinoyl-β-glucuronide, tazarotene, tretinoin
	Antibiotics: clindamycin, erythromycin
	Diverse: azelaic acid, benzoyl peroxide, chemical peels, corticosteroids, dapsone, hydrogen peroxide, niacinamide, salicylic acid, sodium sulfacetamide, sulfur, triclosan
Systemic	Retinoids: isotretinoin
	Antibiotics: azithromycin, clindamycin, co-trimoxazole, doxycycline, erythromycin, levofloxacin, lymecycline, minocycline, roxithromycin
	Hormonal: contraceptives
	Diverse: clofazimine, corticosteroids, ibuprofen, zinc sulfate
Complementary and Alternative Medicines (CAM)	*Achillea millefolium*, amaranth, antimicrobial peptides, arnica, asparagus, basil oil, bay, benzoin, birch, bittersweet nightshade, black cumin, black walnut, borage, Brewer’s yeast, burdock root, calendula, celandine, chamomile, chaste tree, *Commiphora mukul*, copaiba oil, coriander, cucumber, duckweed, Du Zhong extract, English walnut, *Eucalyptus dives*, fresh lemon, garlic, geranium, grapefruit seeds, green tea, jojoba oil, juniper twig, labrador tea, lemon grass, lemon, minerals, neem, oak bark, onion, orange peel, orange, Oregon grape root, patchouli, pea, petitgrain, pine, pomegranate rind extract, poplar, probiotics, pumpkin, resveratrol, rose myrtle, rhubarb, *Rosa damascena*, rosemary, rue, safflower oil, sandalwood, seaweed, soapwort, *Sophora flavescens*, specific antibodies, stinging nettle, sunflower oil, *Taraxacum officinale*, taurine bromamine, tea tree oil, thyme, turmeric, vinegar, vitex, witch hazel, *Withania somnifera* and yerba mate extract
Physical Treatment	Comedone extraction, cryoslush therapy, cryotherapy, electrocauterization, intralesional corticosteroids and optical treatments
